# Automatic Micro-Robotic Identification and Electrical Characterization of Graphene

**DOI:** 10.3390/mi10120870

**Published:** 2019-12-11

**Authors:** Sergio A. Garnica B., Marius Knaust, Sergej Fatikow

**Affiliations:** Department of Micro Robotics and Control Engineering, University of Oldenburg, 26129 Oldenburg, Germany; marius.knaust@uol.de (M.K.); fatikow@uol.de (S.F.)

**Keywords:** microrobotics, graphene identification, graphene characterization, micro four point probe

## Abstract

Micromechanically exfoliating graphene on Si/$SiO_{2}$ substrates is commonplace for graphene researchers, but locating actual graphene flakes on these substrates is a high-effort and tiresome task. The main purpose of this work was to establish a completely automated procedure to identify those graphene flakes with as little human interaction as possible, improving on the limitations of current methods. Furthermore, automatic electrical characterization of the identified flakes was performed. The proposed micro-robotic automation sequence consists of three main steps. To start, a sample surface plane is calculated, based on multiple foci points across the substrate. Secondly, flakes on the substrate are identified in the hue, saturation, and value (HSV) color space, with an implementation to fit the measurement probe, used to avoid undersized samples and adjust the flake orientation. Finally, electrical characterization is performed based on four point probe measurements with the Van der Pauw method. Results of the successfully implemented automation sequence are presented together with flake electrical properties and validation.

## 1. Introduction

2D materials have attracted tremendous attention in recent years from the research community. The possible fields of application range from microelectronics to high sensitivity sensors [1]. The most popular material so far is graphene, a honeycomb lattice of carbon atoms [2].

However, these materials are non trivial to characterize, handle, or process; and many properties are obscured due to the lithographical pre or post processing that is necessary. Normally, lithographical procedures induce contamination and undesired effects on the 2D materials [3]. Other methods [4,5], e.g., chemical vapor deposition (CVD) for large area graphene fabrication and transfer procedures, do not provide the graphene quality that is expected for research tasks [6]. Therefore, micro mechanically exfoliated graphene flakes remain the best alternative for research investigations.

As the preparation of high quality graphene on Si/$SiO_{2}$ substrates via micro-mechanical exfoliation yields unknown flake positions, these have to be located across the whole substrate manually on a high magnification microscope. This exercise requires a high concentration and is extremely time consuming; thus, an approach to diminish this burden would clearly benefit the further exploration of graphene device prototyping.

Micro-mechanical exfoliation research on other 2D materials use similar fabrication and identification methods, via manual optical microscopy imaging search. Therefore, there is a large application field that the method presented in this contribution could be useful for.

This work will focus on a fully automated and reliable way to not only identify, but also electrically characterize graphene. Additionally validation of the flakes will be performed with Raman spectroscopy.

The provided automation sequence will be limited to graphene flakes obtained via micro-mechanical cleavage on a 300 nm Si/$SiO_{2}$ substrate.

## 2. Materials and Methods

### 2.1. Automatic Flake Recognition

Automatic graphene flake identification is of vital importance in the perspectives of future graphene-based devices. Large scale analysis of substrates and the devices produced cannot be performed manually and a reliable methodology for flake localization has to be developed.

Current state of the art image processing techniques for graphene identification [7,8] depend on several defining variables, such as proper background acquisition and/or images with graphene/few-layer-graphene flakes already within them.

Optical microscopy is used to identify 2D materials due to the size of the flakes produced, normally not bigger than tens of micro meters.

Other approaches use lithographic marks on the substrate, and afterwards, find these marks to locate different flakes [9].

Further studies [10] have tried the approach of using a different substrate, e.g., $Al_{2}$/$O_{3}$, in order to enhance the contrast difference, providing an easier way to optically visualize graphene on a surface. This method introduces a non standard substrate, rendering the entry point for any graphene investigation much higher than with the typical Si/$SiO_{2}$, as it is already widely used in the semiconductor industry.

Manual approaches to flake identification with the help of robotic systems have also been investigated before [11]; nevertheless, it is rather focused on the manual modification of graphene flakes. All these methods, although useful in themselves, limit extended complete automatic procedures.

Therefore, an automated procedure for finding flakes, that works on a centimeter scale and which does not have the pitfalls of manually reacquiring background images, could provide a valuable step forward in the implementation of systematic tools to better analyze and develop graphene devices.

### 2.2. Automatic Characterization Approaches

Automatic graphene characterization approaches are mainly based on, either the electrical properties of the graphene itself, e.g., electrical measurements of the conductance [12], or on the intrinsic properties of graphene, atomic force microscopy (AFM) or micro Raman analysis [7].

Additionally, there have been studies related to a thorough characterization of graphene flakes with several methods, non-contact terahertz (THz) time-domain spectroscopy, and Raman Spectroscopy [13]. The main difference from this contribution is the usage of exfoliated graphene flakes for all the measurements, since most of the previous work has been performed on CVD transferred flakes.

The limitation of these approaches is, on one hand, the electrical characterization. It could use lithographic procedures which might modify or contaminate the graphene sample in undesired ways, e.g., by fixed contacts, via dry laser lithography [14].

On the other hand, AFM or THz imaging has to either be integrated into a microscope imaging system or has to be used with a different device, although sample transportation might not be desired.

### 2.3. Nanorobotic Platform

The herein presented nanorobotic platform was based on a robotic microscope objective turret, fitted with 5×, 20×, 50×, and 100× magnification objectives.

**Figure 1:**
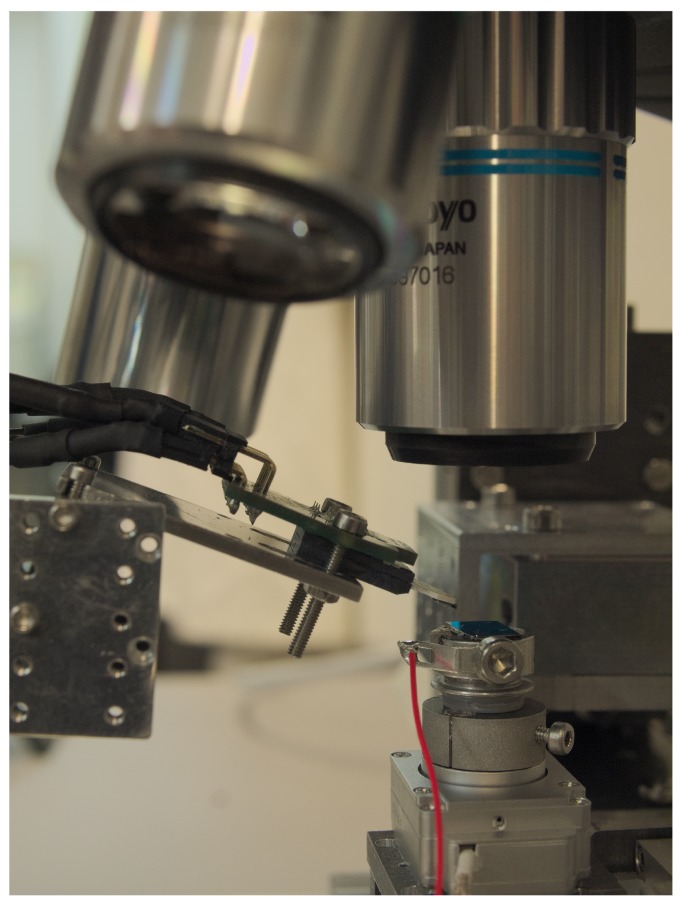
The nanorobotic platform consisting of a Si/$SiO_{2}$ substrate with exfoliated graphene flakes sitting on top of the central sample holder and the micro four point probe chip extended close to the sample surface.

The sample holder stage was composed by three single linear (XYZ) slip-stick driven stages (SmarAct SCL), and the micro four point probe (M4PP) holder stage was assembled from three double linear (XYZ) slip-stick stages (SmarAct SCL). The stages have a range of 2.3 cm with closed loop accuracy of 20 nm. The robotic stages used on these experiments are shown in Figure 1.

Mounted on the sample holder stage *z*-axis, there is a rotation stage (SmarAct SR), which is used as the base for the substrate, the sensor resolution of this stage is 25 μ${}^{\circ }$.

Micro four point probes from Capres A/S (Kongens Lyngby, Denmark) were used, the pitch length between the fingers is 5 μm, as stated by the manufacturer’s data sheet.

The micro four point probe stage is on top of a stack of two goniometers (SmarAct SGO), which helped aligning the M4PP to the substrate surface once.

These robotic stages were used for both coarse and fine positioning, making them ideal for an automated task that requires a large range in the size of the substrate, but also extreme positional accuracy for the measurement of the electrical properties.

**Figure 2:**
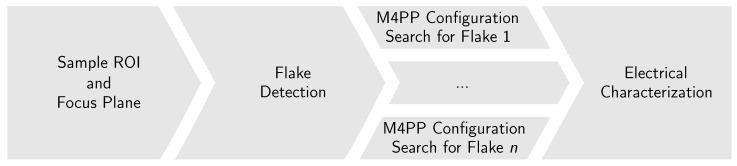
Automation sequence flow diagram, starting from the left side where the region of interest and the sample focus plane are defined. It continues into the flake detection scan of the substrate; as different flakes are found, the best micro four point probe (M4PP) position will be calculated. Finally, the electrical characterization will be performed.

The presented method relies on several steps: calibration made via optical microscope images moving the sample robotic stage to find the substrate’s true focus plane and size; a combination of a rasterized movement along the substrate with a hue, saturation, and value (HSV)-based flake identification algorithm to detect graphene flakes on the surface; and finally, an electrical characterization step, performed with the M4PP. Figure 2 depicts the different automation steps and the implementation order.

All robotic stages, measurement devices, and the vision system are managed by a workstation running Arch Linux operating system. The low level integration of all devices is implemented using a modern C++, which is used from Python to realize the control logic. The computer vision algorithms are realized with the OpenCV library and its data types are converted to Numpy data types by the help of Boost Python.

The presented methodology was tested on graphene flakes obtained via mechanical cleavage procedure on Si/$SiO_{2}$ substrates, with a dioxide layer of 300 nm.

## 3. Results and Discussion

### 3.1. Calibration

This was the first routine performed after the substrate was placed on the sample holder; it was based on images obtained via an optical microscope with a 50× magnification lens.

There are two main purposes of the calibration: the first is to detect the edges of the substrate to acquire the region of interest (ROI) that will be used to search for flakes; the second is to determine the focus plane of the sample’s substrate.

#### 3.1.1. Substrate ROI

An estimation of the substrates dimension is necessary for several reasons. First, it is important to define the region that will be investigated in the search for flakes. Second, if the boundaries are known, it is possible to locate places that are as distant from each other as possible, to calculate the focus plane with a representative sampling along the full area of the substrate.

The main implementation in this step is to determine the edges of the substrate and to find the focus of the substrate next to this edge, which will be explained in more detail in the next section. This task is performed without any previous knowledge of the focus plane, so it works even when the image is not in focus.

The search of the edge was developed as a binary search problem [15]. The algorithm will start by moving a large distance, in the millimeter range, in the direction of the side being searched, until it notices that it has moved over the edge, where there is an all black camera image. In this case it will travel back by half of the previous distance until it can see the substrate again. It will redo the direction change and distance reduction until it finds an image that is half black, as this indicates the physical side of the substrate. This method allows for fast and effective edge identification, especially compared to a naive method of moving smaller distances and checking if the edge has been reached for every step.

After the four sides of the substrate have been found, the ROI to search for graphene flakes is known and the focus plane of the sample can be determined.

#### 3.1.2. Focus Plane

The calculation of a focus plane for the surface of the substrate is necessary to maintain an accurate focus as the robotic system moves the sample under the microscope’s objective. Generally, the sample can not be placed absolutely in parallel to the camera system and the depth of focus of the high magnification objective is limited to 900 nm; the focus is lost when the sample is moved without any height adjustments.

The plane is calculated in the coordinate system provided by the robotic system (SmarAct SCL) on which the sample is placed. For each of the four points next to the edges of the substrate, a precise automatic focus finding procedure is performed and the plane is derived as a linear fit to these points.

The focus searching procedure developed here, was based on optical Newton rings, which were visible on the microscopic images when the sample was positioned 32.5 μm under the focus point of the lens. This pattern appears due to the reflection of the microscope’s light on the surface of the substrate back to the microscope objective, which projects an enlarged image of the objective glass surface. The focus is searched for with the Newton rings and then adjusted by the known offset to the sample, rather than directly for the sample itself, because it produces and image with a consistently high value of variance, even when there is nothing on the surface of the substrate.

The sample is brought to a position roughly 200 μm under the focus, and the Newton rings are then searched by lifting the sample stage closer to the focus of the microscope objective. As the variance of the pattern is much higher than the one of the nearby positions, the variance peak is searched by looking for a significant variance drop with a high slope. Afterwards, a sweep along the peak is performed and the focus position is calculated with a Gaussian fit.

### 3.2. Sample Scanning

As it is very time consuming to search the 1.1 cm×0.7 cm substrate with a view-field of only 224.4 μm×119.46 μm, with the 50× magnification objective manually for flakes, a reliable flake detection is indispensable. Further, pinpointing a location on the identified flakes for the electrical characterization is of great importance.

The sample is scanned in the previously defined ROI (Section 3.1.1) line by line, with alternating directions. The frames overlap, so every flake is seen at least once completely. Duplicates will be sorted out afterwards.

For each frame an asynchronous flake detection is executed, as described next.

#### 3.2.1. Flake Detection

The methodology developed, and presented here, is based on the hue, saturation, and value (HSV) color space. This is one of the most common cylindrical coordinate representations of points in a RGB color model [16].

HSV color space offers some critical advantages that fit this problem well. It allows us to select a single continues range for each parameter. The *hue* is used to distinguish graphite from the substrate and the *saturation* is directly proportional to the amount of layers, which suits it perfectly to select graphene, single layer graphite. The *value*, also called brightness, is less important for identification, since it might change due to the not perfectly homogeneous illumination and focus. Hence, selecting a wide HSV range makes the procedure less prone for errors in different light angles and slight off focus positions.

The HSV ranges were empirically investigated and are only valid for graphene flakes consisting of up to three layers on a substrate with 300 nm $SiO_{2}$. Different dioxide sizes or two dimensional materials could be also integrated with relatively little effort. These HSV ranges depend on the illumination intensity of the microscope light used and will change depending on the system used; re-calibrating the range is not complicated as target flakes can be imaged under the desired system, and from these images a rough range can be extracted for any microscope and light condition used, given that the light state does not change during operation.

**Figure 3:**
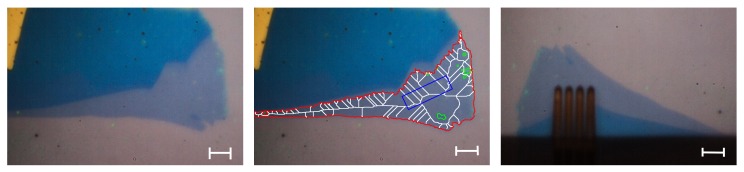
Automation sequence images provided by the method presented in this paper. (**Left**) Flake detected via the HSV range after the focus plane was established by the system. (**Middle**) Flake detected with an overlay of (**red**) flake contour; (**green**) holes detected inside of the flake contour; (**white**) skeletonized version of the flake contour minus the holes; (**blue**) best position found for the micro four point probe investigation, including the orientation. (**Right**) rotated flake with micro four point probes during the electrical characterization. The scale bar represents 10 μm and is valid for the left and center images.

As a possible flake is recognized, the contour is extracted from the image. An example detected flake is shown on the left image on Figure 3. If the region contains pixels that are outside of the defined HSV range, e.g., contamination on the flake, they get marked as holes.

Additional post processing helps to filter the results by size and eliminates the same flake being detected twice, due to the acquisition of overlapping images. The latter is achieved by checking for intersections between all the results in the global coordinate system and picking a representative candidate, based on the best fit for the M4PP (described in the next section).

#### 3.2.2. M4PP Configuration Search

Once a flake is identified via the HSV range set in the previous section, it is necessary to investigate whether this flake is appropriate for the electrical characterization.

It is important to decide if the flake is large enough to fit the M4PP, in order to perform the measurement. The M4PP is modeled as a rectangle that should be fitted inside the detected contour, a polygon. The center image of Figure 3 shows an example of the patterns created based on the algorithm explained below.

Fitting a polygon inside a polygon is a non trivial problem to solve, and could be time consuming if implemented naively; e.g., a brute-force try and error approach on every possible pixel position. Another complexity level is added when adjusting the orientation of the M4PP is considered.

In the proposed solution is the usage of additional requirements, derived from the electrical characterization, to reduce the search space. Particularly, the M4PP should be placed as far from the borders of the flake as possible, since edge and boundary effects can affect the measurement. This can be directly translated to a reduction of possible positions for the M4PP, to the center of the flake’s polygon.

This reduction is archived by a skeletonized version of the contour, created according to [17]. As the contour skeleton represents the farthest distance from the edges of the flake to the center, rotations along the skeleton are tried. If the M4PP is covered by the flakes polygon, the configuration is considered a possible candidate. Afterwards, all candidates are ranked against each other by the steps it takes to scale them up, until they intersect with the enclosing polygon. The position with the maximum amount of steps is chosen for the measurement.

Additionally, it could be possible to think of pruning the skeleton by half of the smaller side of the rotated enclosing rectangle of the M4PP’s polygon, to further optimize the procedure. In this study it was not necessary, since the the fitting jobs were run in parallel to the image acquisition, which is the dominant factor regarding the runtime.

Finally, an image of the surrounding box of the flake is saved as a reference for the M4PP measurement.

### 3.3. Four Point Probe Electrical Characterization

One of the easiest paths to obtain the electronic properties of a sample, is to attach two wires to the sample, let a current (*I*) run through it and measure the voltage drop (*V*).

However, a two-point measurement will include serial, spreading, and contact resistances. A more sophisticated measurement is to contact the sample with four independent wires, and measure the voltage drop across two of these, while passing a current through the two other wires. This will eliminate all wire and contact resistances, since the supplied current is the same as in a serial circuit, and the voltmeter has a very high internal resistance. The sheet conductance then becomes σ=I/c·V, where *c* is the geometrical correction factor [9,18].

A semiconductor device analyzer (B1500A, Keysight, Santa Rosa, CA, USA) was used as the sourcing and measuring equipment during all the experiments. This device was selected because of the flexibility that it provides when setting a measuring step, as different channels can be used either in current or voltage mode, so the electrodes act either as sources or monitor channels.

#### 3.3.1. Van der Pauw

Limiting factors on the positioning system produce some geometrical and positional errors of the electrodes that have to be compensated when performing a M4PP measurement.

**Figure 4:**
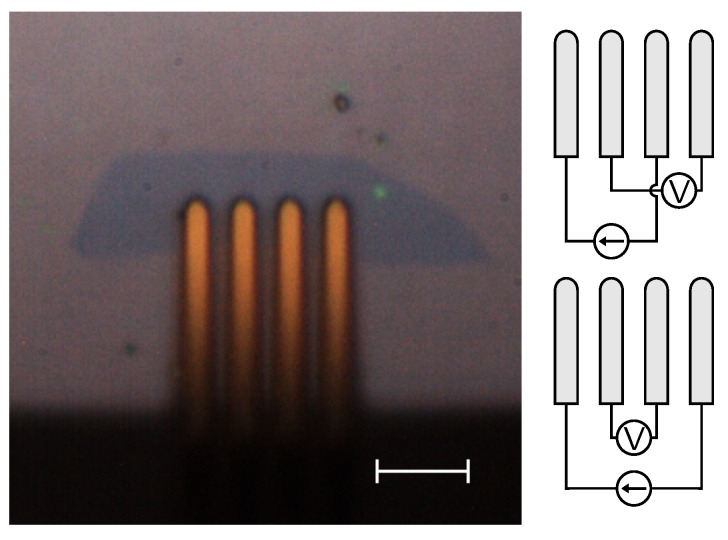
(**Left**) Flake used for electrical characterization shown in Figure 5. (**Right**) Dual configuration used for the measurement of the electrical properties of the flakes. The scale bar represents 10 μm.

A dual configuration measurement [19], as shown in Figure 4, corrects for these geometrical errors caused by the finite sample size and the static error on the correction factor.

#### 3.3.2. Centering and Approximation

The practical implementation of the automatic measurement has to take into account the information obtained from the flake detection procedure.

The orientation of the flake has to be adjusted to the previously determined position, while taking care to bring the flake back into the microscope’s field of view. The latter is achieved by determining the rough position by a rotation of the position vector around the predefined rotational axis. This position is then corrected by refocusing, and template matching a rotated and masked version of the previously acquired image of the flake.

Afterwards, the M4PP is brought into focus with the flake underneath it. The substrate is then slowly moved towards the M4PP, while a continuous measurement in the M4PP is performed. As soon as the resistance drops below a certain threshold, the flake is in contact with the M4PP. Afterwards a dual configuration sweep measurement is conducted on the flake.

**Figure 5:**
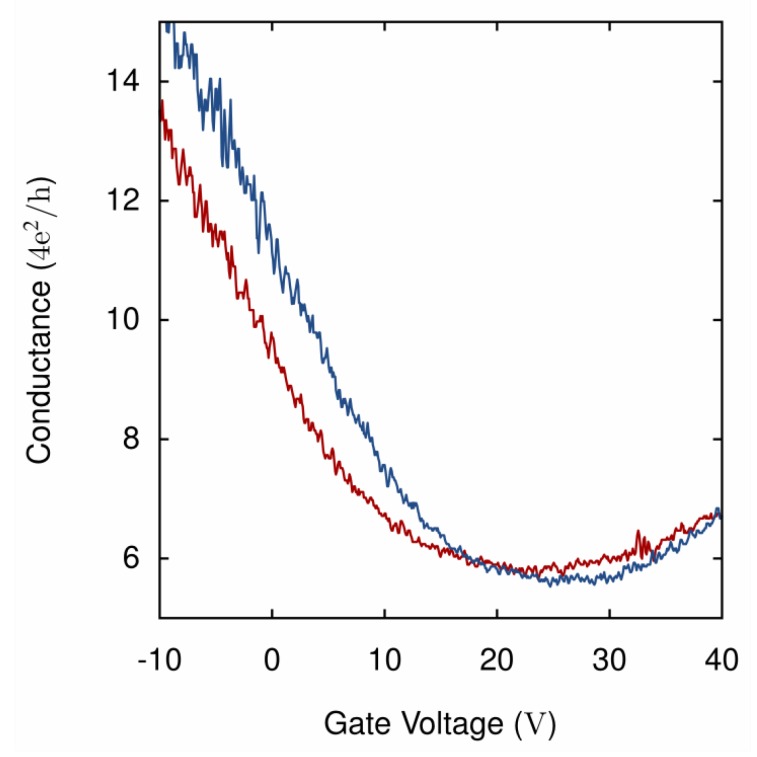
Conductance as a function of the applied gate voltage result, for the flake depicted in Figure 4. Red shows the increasing gate sweep and blue is the decreasing gate sweep.

Typical results for the electrical characterization can be seen in Figure 5; this graph shows the conductance as a function of the gate voltage of the graphene flake in Figure 4.

The electrical characterization shows how the flake charge neutrality point is located roughly between 20 and 30 V, which indicates doping on the flake. It is also possible to see the hysteresis of the two different sweep directions of the gate voltage, as the increasing gate sweep is slightly different to the decreasing gate sweep.

The conductance value states that the flake most likely is not single layer graphene but few layers of graphene, as the charge neutrality point of single layer graphene should result in a much lower conductance value.

### 3.4. Automation Runtimes

As the proposed automation sequence was evaluated, speed was second to accuracy in flake finding. Bottlenecks arouse not from the constantly processing images, but from the wait that had to be implemented due to the vibration of a slip-stick driven stage.

On a 1.1 cm×0.7 cm substrate, finding the focus plane, including the edges of the sample, took around 5 min. Searching for flakes within the automatically calculated ROI took about 1.5 h. When performing the electrical characterization the average movement to the identified flake position will depend on the calculated angle; the bigger the angle, the longer it takes to reach that position. The times will vary between 10 and 30 s. The semiconductor device analyzer will take around 13 s to perform a full −40 V–40 V and backwards sweep with both configurations on the M4PP.

In conclusion, the automatic identification and electrical characterization procedure will take less than 2 h for a substrate with about 1.0 cm side length.

### 3.5. Validation

In order to corroborate that the M4PP did not damage the flake during the electrical measurement, Raman spectroscopy of one of the measured flakes was obtained, excitation wavelength: 514 nm; beam power 2 mW.

**Figure 6:**
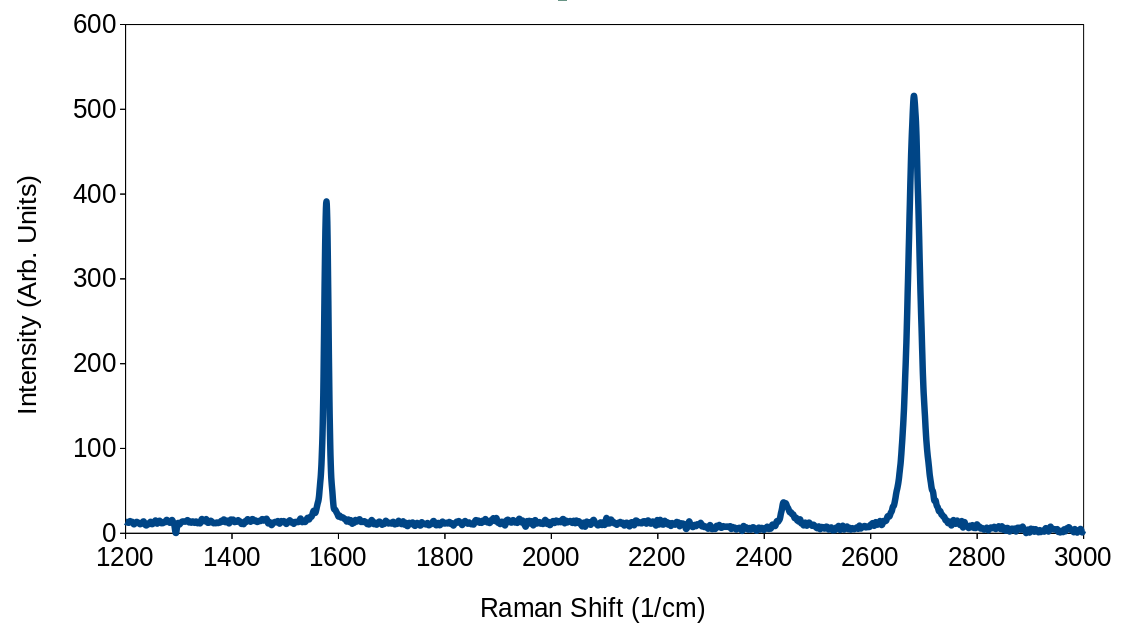
Raman spectra of one of the graphene flakes after the electrical characterization via the M4PP.

It is possible to observe from the spectra in Figure 6 that the flake was not single layer, as the peaks ratios have very similar intensities; nevertheless, it was a few layers of graphene, as the G peak at around 1550 cm^−1^ is not much higher that the 2D peak at around 2700 cm^−1^.

Also noteworthy, is the lack of a peak at around 1300 cm^−1^, which would indicate that significant defects were introduced on the flake; as this is not the case, it is safe to assume that the procedure did not structurally modifiy the flake under study.

## 4. Conclusions

A totally automated micro robotic graphene identification and electrical characterization methodology is presented. The procedure improves on the limitations of other robotic, state-of-the-art graphene identification methods, using HSV color space values to detect graphene flakes on a 300 nm $SiO_{2}$ substrate.

It also selects suitable flakes for later experiments, based on the available area to probe the flake with a micro four point probe. Additionally, it selects the best angle configuration for the electrical characterization, so edge and boundary effects are reduced as much as possible. System-provided results were shown and described in detail.

Future work will focus not only on the characterization of the 2D materials, but also on the systematic functionalization or modifications of graphene flakes. This contribution serves as the base for a series of 2D materials-scanning probe microscopy studies. These will be performed with an improved robotic platform when two independent end effectors are added.

## Abbreviations

The following abbreviations are used in this manuscript:

HSV	Hue, Saturation, and Value
M4PP	Micro Four Point Probe
ROI	Region of Interest
